# Botulinum toxin as a treatment for short bowel syndrome in rats^1^

**DOI:** 10.1590/s0102-865020190070000005

**Published:** 2019-09-16

**Authors:** Isabela Cristina de Souza Marques, Stefânia Bovo Minto, Mariane Quaglio Marques, Juliana Ribeiro, Paola Castro Moraes, Lourenço Sbragia, Sérgio Britto Garcia

**Affiliations:** I Fellow PhD degree, Postgraduate Program in Pathology, Department of Pathology and Legal Medicine, School of Medicine of Ribeirao Preto, Universidade de São Paulo (USP), Ribeirao Preto-SP, Brazil. Conception, design, and scientific content of the study; II Fellow PhD degree, Postgraduate Program in Pathology, Department of Pathology and Legal Medicine, School of Medicine of Ribeirao Preto, USP, Ribeirao Preto-SP, Brazil. Histopathological examinations, statistical analysis; III Graduate student, Department of Pathology and Legal Medicine, School of Medicine of Ribeirao Preto, USP, Ribeirao Preto-SP, Brazil. Design and scientific content of the study; IV Fellow PhD degree, Postgraduate Program in Veterinary Surgery, Faculty of Veterinary Medicine, Universidade Estadual Paulista (UNESP), Jaboticabal-SP, Brazil. Design and scientific content of the study; V PhD, Assistant Professor, Faculty of Veterinary Medicine, UNESP, Jaboticabal-SP, Brazil. Conception, design, intellectual and scientific content of the study; VI PhD, Associated Professor, Department of Surgery and Anatomy, School of Medicine of Ribeirao Preto, USP, Sao Paulo-SP, Brazil. Conception, design, intellectual and scientific content of the study; critical revision; VII PhD, Full Professor, Department of Pathology and Legal Medicine, School of Medicine of Ribeirao Preto, USP, Sao Paulo-SP, Brazil. Conception, design, intellectual and scientific content of the study; critical revision; final approval

**Keywords:** Short Bowel Syndrome, Botulinum Toxins, Ileum, Rats

## Abstract

**Purpose:**

The denervation of the intestine with benzalkonium chloride (BAC) reduces mortality and improves weight gain in rats with short bowel syndrome (SBS). Nevertheless, translating these promising findings from bench to bedside is not feasible because BAC promotes peritonitis and irreversible denervation which may be followed by an uncontrolled dilatation of the viscera. The use of botulinum toxin (BT) instead of BAC to achieve the denervation of the remaining small intestine in SBS could be an interesting option because it leads to a mild and transient denervation of the intestine.

**Methods:**

Here we evaluated the effects of the ileal denervation with BT in rats with SBS by verifying the body weight variation and intestinal morphological parameters. Four groups with 6 animals each were submitted to enterectomy with an ileal injection of saline (group E) or BT (group EBT). Control groups were submitted to simulated surgery with an ileal injection of BT (group BT) or saline (group C - control).

**Results:**

We observed that the treatment of the remaining ileum with BT completely reversed the weight loss associated to extensive small bowel resection.

**Conclusion:**

This may provide a new promising approach to the surgical treatment of SBS.

## Introduction

Short bowel syndrome (SBS) is an intestinal failure resulting from an insufficient absorption surface of the intestine, usually due to surgical removal of more than 50% of the intestine length. The hallmark symptoms of the SBS are malnutrition and weight loss. Parenteral nutrition, as well as several clinical and surgical procedures have been used on the patients with SBS and in experimental settings. Nevertheless, only limited success has been achieved so far. In fact, morbidity and mortality rates remain disappointedly high in patients with SBS. “The development of new and innovate therapy designed to amplify the intestinal adaptation after extensive resections is required to improve and save human life” has been proposed by Longshore, in 2009^1^. In regard to this, it has been observed that the myenteric denervation of the remaining small intestine was able to reduce mortality in rats with SBS^2^. In sequence we have confirmed these findings and described that the clinical improvements were due to a massive increase of the intestinal absorptive surface in rats with SBS with the BAC denervation^3^. We have also found similar results with the denervation of the jejunum^4^. The effects of BAC denervation in SBS were further confirmed by other authors^5^. Nevertheless, despite the encouraging preliminary results and the usefulness of the BAC experimental model for the comprehension of the role of the enteric nervous system in intestinal adaptations after extensive resections, translating these promising findings from bench to bedside is not yet a feasible task. In fact, it would be reckless to perform such experiments in humans because BAC promotes peritonitis with further risk of pseudo-obstruction, as well as irreversible denervation followed by uncontrolled dilatation of the viscera^6^. In this aspect the use of botulinum toxin (BT) instead of BAC as a way of achieving the denervation of the remaining small intestine in SBS could be an interesting option because it leads to a mild and transient denervation of the intestine^7^. Here, we evaluated the effects of the ileal denervation with BT in rats with SBS by verifying the body weight variation and intestinal morphological parameters.

## Methods

The study was approved by an institutional ethics committee for animal experimentation (Protocol 016529/2017 - UNESP).

Twenty-four Wistar rats (weight range 50-70g) were randomly divided into four groups with 6 animals each, as follows: 1) E Group - enterectomized with an ileal injection of saline; 2) EBT Group - enterectomized with an ileal injection of BT; 3) BT Group - simulated surgery with an ileal injection of BT and 4) C group - simulated surgery, with an ileal injection of saline.

The enterectomy consisted of 80% resection of the small intestine length with end-to-end anastomosis and the remaining small intestine consisted of equal lengths of jejunum and ileum, as described^3^. For the simulated surgery, the intestine was sectioned without resection and a re-anastomosis was performed. The application of either BT or saline was performed after the re-anastomosis. 100 units of Botox^®^ (BT) were diluted in 2ml of saline solution and each animal received 10U of BT through 5 microinjections into the ileal wall, using a 25-gauge sclerotherapy needle. The microinjections were performed in a semi-circumferential manner within the wall of the ileum, starting 1.5 cm above the ileo-cecal junction infiltrating the wall in oral (proximal) direction for 3.0 cm. Anesthesia was performed with ketamine, 50 mg/mL (Ketamina^®^ - Pfizer - Sao Paulo, SP-Brazil) and xylazine, 10 mg/mL (Rompum^®^- Bayer - Sao Paulo, SP- Brazil). Dipyrone was administrated in the post operatory period, as well as enrofloxaxine for 3 days.

The animals received standard diet and water *ad libitum.* Weight was assessed before the beginning of the experiment (initial weight), then weekly and at the end of the experimental period (30 days after the surgical procedures). The animals were euthanized by CO_2_ inhalation. Small intestine weight, length and diameter were measured. Annular intestinal tissue samples (as rings) were collected from each animal at the middle of the remaining jejunum and ileum, encompassing the entire thickness of the small bowel wall. The samples were fixed in buffered formalin, embedded in paraffin and cut serially, maintaining the annular orientation. Ten sections with 5μm each were obtained per animal, stained with H&E and used for the morphometric study of the intestinal mucosa, using an Imaging System software (Kontron Image Analysis KS 300, of Kontron ElektronikGmbh, Image Analysis Division-Augsburg, Germany) in conjunction with a Zeiss microscope fitted with a video camera that transferred the images from the microscope to the computer screen, in which the parameters were delimited. The analysis was made under 100x magnification using specimens in which the villi and the crypts were perpendicular to the *muscularis mucosae.* The following measurements were obtained: (a) Villus height (from the top of the villus to the villus-crypt junction), (b) crypt depth and (c) muscular layer thickness. The measurements were expressed in microns (averages). The results were expressed as mean ± standard error. Means from the morphometry data and body weights were both analyzed by ANOVA test, with the level of significance set at 5% and the Statistical Analysis System for Windows (SAS 8.01, SAS Institute Corporation 1999-2000, Cary, NC, USA).

## Results

**Figure 1:**
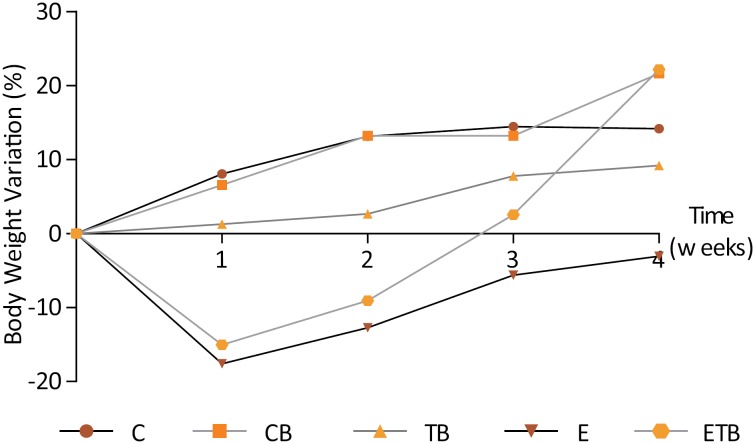
Weight variation during the experimental period (%). Comparison of the weight gain of the animals belonging to the study groups. Statistical test used: One-way ANOVA. P = 0.0421 (GraphPad Prism 5.0).

No differences in food intake were observed among the experimental groups, and at the end of the experimental period all the animals were healthy. Both the surgical procedures and the BT injections were well tolerated, and no side effects were seen in the animals. At the end of the experimental period, the group E, that underwent an enterectomy, presented marked weight loss (P<0.05). Interestingly, the EBT group showed an increase in weight similar to that of the control animals (Fig. 1).

**Figure 2:**
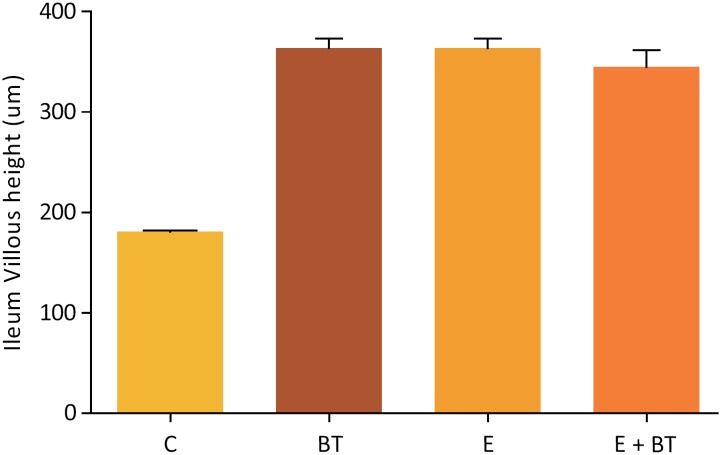
Ileum villous height (μm). Comparison of the Ileum Villous height, in μm, of the animals belonging to the study groups. Statistical test used: One-way ANOVA. P<0.0001 (GraphPad Prism 5.0).

**Figure 3:**
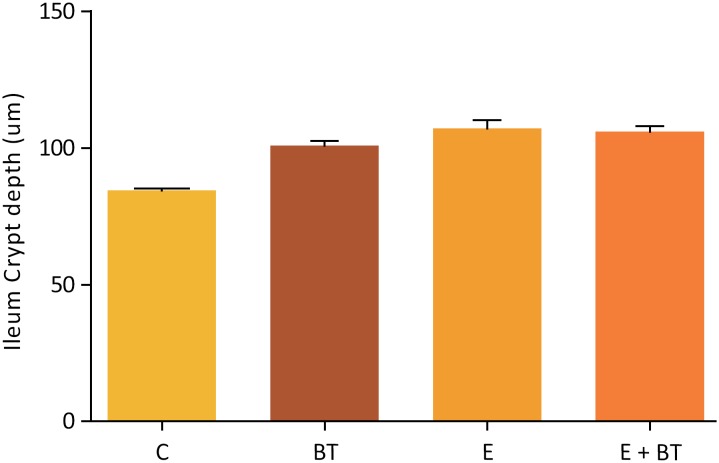
Ileum Crypt depth (μm). Comparison of the Ileum Crypt depth, in μm, of the animals belonging to the study groups. Statistical test used: One-way ANOVA. P<0.0001 (GraphPad Prism 5.0).

**Figure 4:**
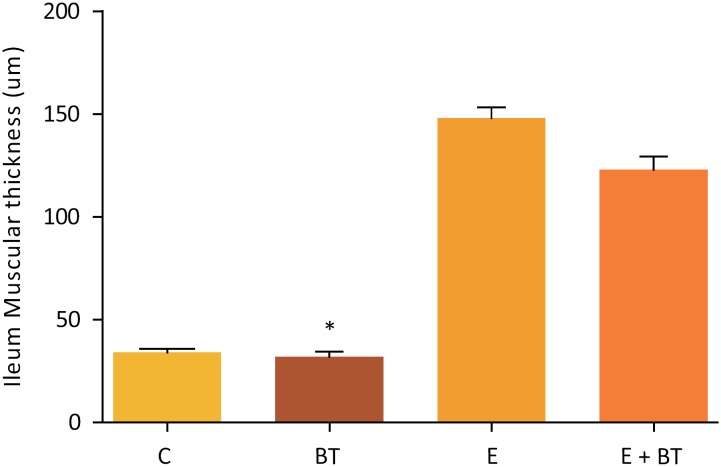
Ileum Muscular thickness (μm). Comparison of the Ileum muscular thickness, in μm, of the animals belonging to the study groups. Statistical test used: One-way ANOVA. P<0.0001 (GraphPad Prism 5.0). The BT group showed a decrease in this factor when compared with the groups E (P= 0.0050) and E + BT (P=0,0050) (Mann-Whitney test).

**Figure 5:**
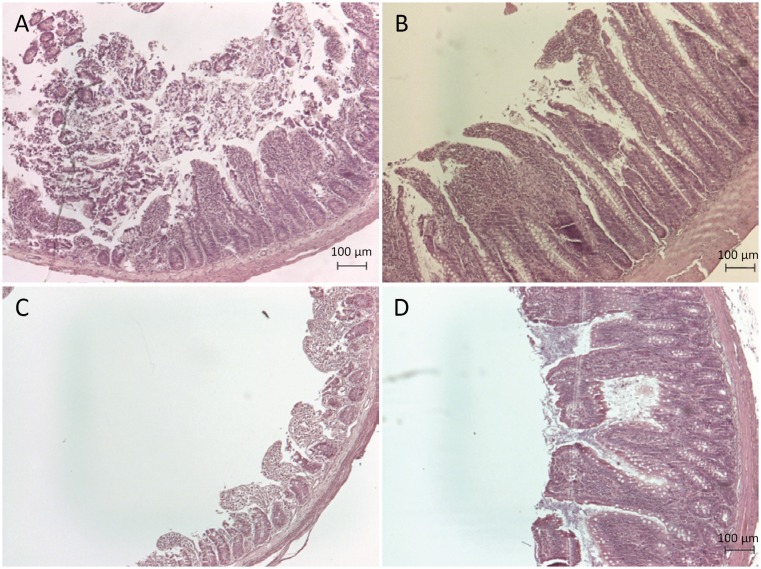
Representative histological sections of the ileum of experimental groups. **(A)** Control animals. **(B)** Animals with intestinal resection exhibit an increase of the villus size. **(C)** and **(D)** BT-treated animals also showed an increase of the villus size.

Macroscopically, the intestinal remnants did not change in length among the experimental groups (data not shown) but the animals from the Groups BT and EBT showed a noticeable increase in the ileum diameter in the segment submitted to the BT application (which was not observed in the other groups). The histopathological and morphometric analysis revealed mucosal hyperplasia in the ileums of all the animals from groups E, EBT and BT, with statistically significant increase in mucosa, villi height, crypt depth and muscular thickness (Figs. 2 to 5). Nevertheless, no statistical difference was found among the three experimental groups.

## Discussion

We observed in all the operated animals that the treatment of the remaining ileum with BT completely reversed the weight loss associated to extensive small bowel resection. The present findings are similar to previous reports in the literature regarding the beneficial effects of denervation with benzalkonium chloride in rats submitted to experimental SBS^3^ ^,^ ^4^.

In response to extensive intestinal resection, the remaining small bowel, mainly the ileum, develops adaptation mechanisms to compensate the loss of absorptive mucosal surface, with hyperplasia of the mucosa and increase in cell proliferation that has been well described by some authors^1^ ^,^ ^8^ ^,^ ^9^. Denervation with BAC causes a further increase in the mucosal hyperplasia in rats that had been observed, which may, at least, partially explain the improvement in weight gain in this experimental model^3^. Strikingly, here we observed that the BT application also caused an improvement of weight gain but it was not associated to any further increase in the intestinal absorptive surface area, apart from the expected adaptive response to the intestinal resection. Thus, it is likely that the beneficial effects of the BT application may be due to an eventual reduction in peristaltism, which could facilitate the absorption of nutrients through the intestine. This hypothesis should be investigated in the future and is reinforced by the previous report that BT reduces ileal peristaltism by targeting cholinergic neurons, as observed in botulism^10^.

Taking into account that in SBS the remaining intestinal absorptive and digestive capacities are increased^1^, the hypothesis that the BT-denervation could influence the absorptive capacity by unknown mechanisms could not be ruled out and should be investigated in future mechanistic studies using the experimental model described here.

Since BT injected into the ileal wall clearly improved the weight gain of rats with SBS, this study opens the gate to a minimally invasive and non-expensive treatment for patients with SBS. The major advantage of the use of BT instead of BAC to achieve intestinal denervation is that the applicability of BT for human patients is safer. BT has been successfully used in human clinical gastroenterology in various situations. For example, esophageal BT injections are beneficial in improving some esophageal motility disorders, like achalasia^11^ ^,^ ^12^ and anal fissures^13^. Nevertheless, despite the favorable experimental results, more data are required on the safety and effectiveness of this technique before solid conclusions can be reached. The eventual use of this procedure for human patients with SBS must take into account some possible collateral effects, such as induction of severe bacterial overgrowth due to luminal stasis.

## Conclusion

The botulinum toxin application may provide a new promising approach to the surgical treatment of SBS.
